# Longitudinal Radiographic Study of Cranial Bone Growth in Young Cheetah

**DOI:** 10.3389/fvets.2019.00256

**Published:** 2019-07-30

**Authors:** Gerhard Steenkamp, Martin J. Schmidt, Paul J. van Staden, Marthàn N. Bester

**Affiliations:** ^1^Department of Zoology and Entomology, Faculty of Natural and Agricultural Sciences, Mammal Research Institute, University of Pretoria, Pretoria, South Africa; ^2^Department of Companion Animal Clinical Studies, Faculty of Veterinary Science, University of Pretoria, Pretoria, South Africa; ^3^Department of Veterinary Clinical Sciences, Small Animal Clinic-Surgery, Justus Liebig University Giessen, Giessen, Germany; ^4^Department of Statistics, Faculty of Natural and Agricultural Sciences, University of Pretoria, Pretoria, South Africa

**Keywords:** cheetah, skull, facial growth, molar, mandible, radiograph, palatitis

## Abstract

Focal palatitis (also known as focal palatine erosion) is thought to be a developmental disease, specifically of cheetah in captivity raised on a commercial diet. The lack of chewing is thought to cause the mandibular molar to change angulation, contacting the palate and causing the lesions. We followed the development of five captive cheetah cubs, born within 2 weeks of each other, at the same facility. This longitudinal study followed the cubs cephalometrically from 7-months-old to 25-months-old. Of each cub we made a lateral and dorsoventral radiograph at 7, 13, 20, and 25-months-old. For each radiograph at each age, a predefined series of measurements were made including the angle of the molar. The latter was measured as the angle of the bisecting line running through the apex of the caudal molar root intersecting with a line drawn at the ventral margin of the mandible. The results confirmed that the cheetah skull and neurocranium follows the same neural growth pattern as has been described for other mammalians. Similarly the maxillofacial component follows the same somatic growth as seen in all mammalians excluding humans and non-human primates, where a pubertal growth spurt is present. Finally the angle of the mandibular molar at 7 months differed significantly from the angle at the other ages, however there were no statistical difference in the angulation of the molar after eruption (13 months and older ages). In these five cheetah the lack of chewing (as seen in captivity with a commercial or meat only based diet) did not alter the angulation of the mandibular molar, nor did the mandibular molars super erupt in these patients at 25-months-of-age.

## Introduction

Focal palatitis FP [formerly focal palatine erosion (FPE)] (1) lesions in cheetahs have been described as a developmental condition (2). It is characterized by erosion of the oral mucosa and by a localized osteomyelitis of the palatine bone in advanced stages of the disease (3). The pathophysiology of FP is not fully understood. The original description of FPE concluded that the molar supposedly erupts higher (super eruption) than normal and changes its angulation mesially thereby contacting the palate and causing the lesions. The change in angulation of the molars was ascribed to a captive diet without tough texture thereby shortening the duration and intensity of the chewing action in these animals (3). The latter opinion has been challenged and an alternative pathogenesis has been put forward by Steenkamp (1). It is believed that the normal indentations occurring on the palate of cheetah may trap food or other foreign bodies. The ensuing infection and associated inflammation may then ultimately cause localized osteomyelitis, which may progress to oro-nasal fistulas at some of these palatal indentations—often associated with the mandibular molar's indentation, palatal to the maxillary 4th premolar tooth (4).

Whereas radiographic and tomographic imaging methods were used to study the general morphology of dog skulls (5, 6) and pigs (7), data about cranio-maxillofacial growth in the ontogeny of large felids are not available. Once such a longitudinal cephalometric description exists for the cheetah, it will be possible to utilize the observed growth pattern in order to document real time changes taking place.

In this study we document the growth of the cranio-maxillofacial complex in cheetah from 7 to 25-months-of-age using repetitive radiographic examination. General growth trajectories as well as the eruption of the mandibular molar in the cheetah in particular are investigated, in order to clarify the developmental aspect of FP.

## Materials and Methods

Five captive bred cheetah cubs, all born within 2 weeks from one another and belonging to two different litters were used for this study. They originated from the Ann van Dyk Cheetah Center 30 km northwest of Pretoria. The diet of the cubs consisted of milk from the females up to 8 weeks old. A diet consisting of boiled minced chicken supplemented with 30 g of a commercial cat food, egg, milk, vitamin, and mineral supplements was then introduced and fed until the cubs were 3 months-old. From then on, the diet consisted of chicken or horse meat, supplemented with a vitamin, and mineral powder.

From 7-months-of-age they were anesthetized according to the institution's protocols, on average every 6 months (6, 7, and 5 months) until they were 25-months-of-age. The latter age was selected as the animals are thought to be fully grown by 24-months-of-age. Furthermore, at 24-months-old, the animals are often divided into breeding groups or animals that will be sold and no further access to the animals was possible. At each of these anesthetic events, a lateral and dorsoventral skull radiograph was made. A 10 cm radiographic guide was placed on the detector plate with the skull to be radiographed, at a level central to the skull. This was done for future use in correcting for any image distortion that may take place.

Radiographic images were made using a Shimadzu MD 100 x-ray machine (Axim, Corporate Park South, Midrand, South Africa) and a Carestream Vita CR computed radiography (CR) system (Lomaen Medical, Edenvale, South Africa) at a film focal distance (FFD) of 100 cm. Detailed exposure settings are summarized in Table 1. The radiographic system used to acquire the pictures of the first three visits was not available for the final evaluation. We were not able to source a similar machine and therefore a digital direct radiography (DR) (and not CR) system was used (Leonardo DR Systems, OR Technology, Rostock, Germany).

**Table 1:** The exposure factors used to radiograph the skull of five growing cheetah (kV, kilovolts; mAs, milliampere seconds).

**Date**	**kV**	**mAs**	**System**
13/12/2011	50	4	Carestream CR
05/06/2012	55	8	Carestream CR
23/01/2013	57	10	Carestream CR
04/06/2013	46	3	Leonardo DR

The study was approved by the University of Pretoria's Animal Ethics Committee (Project: EC062-11). All the measurements were made utilizing specific software (KPACS, IMAGE Information Systems Ltd., London, United Kingdom) and recorded in a spreadsheet developed for this purpose.

**Figure 1:**
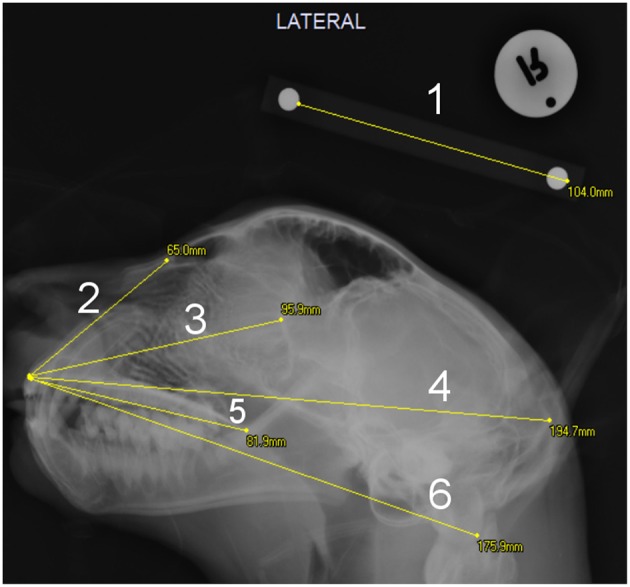
Extent of measurements on the lateral skull radiographs: 1- The 100 mm scale barr; 2- Facial length2; 3- Facial length1; 4- Total skull length; 5- Palate length; 6- Condylobasal length.

**Figure 2:**
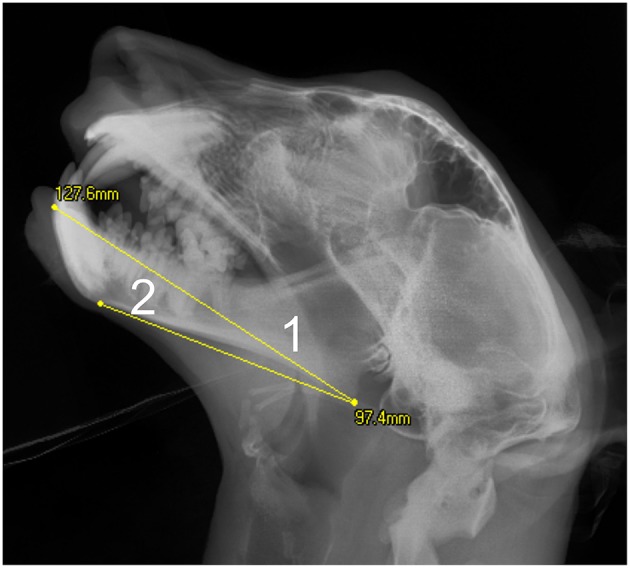
Extent of measurements on the mandible taken from the lateral skull radiographs: 1- Mandibular length1; 2- Mandibular length2. On this cropped image the 100 mm scale bar is not visible.

**Figure 3:**
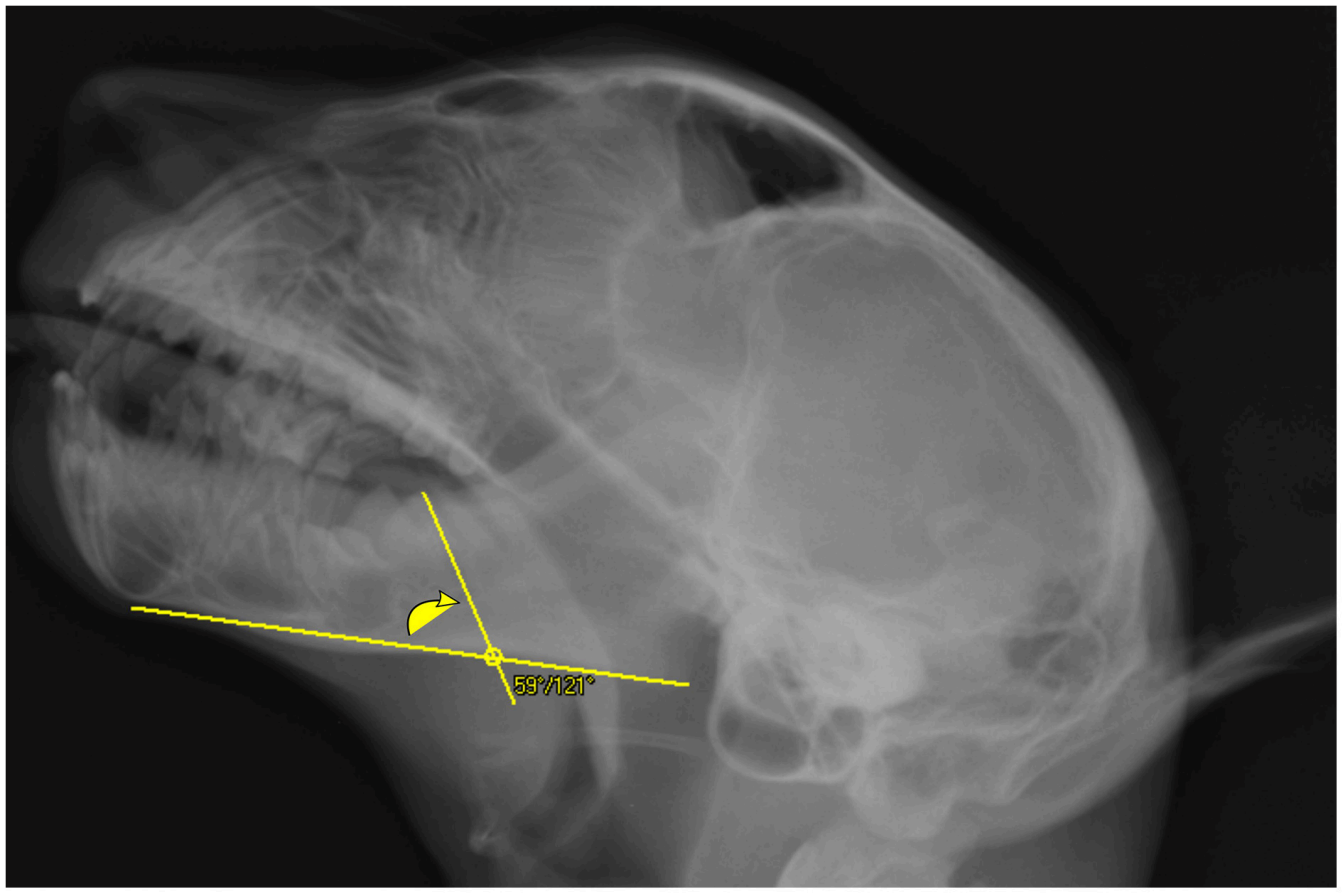
Depiction of a line drawn through the caudal cusp and apex of the molar tooth crosses the mandibular length2 line on the lateral skull radiograph of a cheetah cub. The inside angle where these two lines cross was measured. On this cropped image the 100 mm scale bar is not visible.

**Figure 4:**
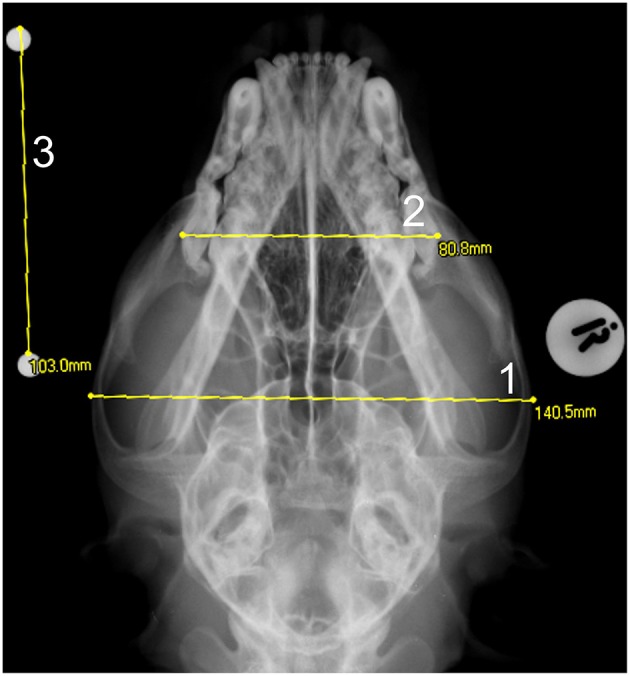
A dorso-ventral skull radiograph of a cheetah cub showing the measurement of the: 1- maximum skull width and 2- the maximum maxilla width. A 100 mm scale bar was placed at a height equal to the middle of the skull in order to quantify the enlargement factor -3.

To investigate longitudinal growth of the skull, with main focus on the mandible and maxilla, six cephalometric measuring points were used based on previous morphometric and cephalometric work (6, 8–11) and adapted for this study. Linear connection of these points characterized growth trajectories and their changes over time. All measurements are described in Table 2 and illustrated in Figures 1–4. The specific landmarks used for this longitudinal skull growth study were the following:

**Inion:** Central surface point on external occipital protuberance.
**Infradentale:** Most antero-superior (rostro-dorsal) point on the labial crest of the mandibular alveolar process.
**Menton:** Lowest point on the lower border of the mandibular symphysis.
**Nasion:** Junction on medial plane on the left and right nasofrontal sutures.
**Prostion:** Rostral end of inter-incisive suture, located between roots of upper central incisor teeth.
**Zygion:** Most lateral point of zygomatic arch.

**Table 2:** Measurements used to describe the cephalometrics of development in the skulls of five growing cheetah cubs.

**Measurement**	**Description**
Condylobasal length	Prosthion to caudal border of occipital condyle
Palatal length	Prosthion to caudal nasal spine of palatine bone
Total skull length	Prosthion to Inion
Facial length1	Prosthion to middle of the ethmoid
Facial length2	Prosthion to Nasion
Neurocranium	Caudal border of occipital condyle to middle of ethmoid
Skull width	Zygion to zygion
Maxilla width	PM4 alveolar margin to PM4 alveolar margin
Mandible length1	Infradentale to caudal condyle
Mandible length2	Menton to caudal condyle
Mandibular height	Height of mandible caudal to the mandibular molar perpendicular to the mandibular plane
Molar height	Caudal margin from cemento enamel junction to caudal cusp
Molar angle	The angle of a line drawn from the caudal cusp through the caudal root apex and its intersection with the mandible length1

The distances measured as described in Table 2, were then used to record a number of indices (Table 3). From the length and width measurements, growth rate was determined using the formula: $\text{Growth rate}=\frac{\text{mm measured}}{\text{number of months of time interval}}.$

**Table 3:** Indices calculated for the five cheetah cubs, based on linear skull measurements.

**Index**	**Calculation**
Palate1	Palate length/Condylobasal length
Palate2	Palate length/Total skull length
Facial1	Facial length1/Condylobasal length
Facial2	Facial length1/Total skull length
Facial3	Facial length2/Condylobasal length
Facial4	Facial length2/Total skull length
Facial5	Facial length1/Neurocranium
Facial6	Facial length2/Neurocranium
Max-Skull width	Maxilla width/Skull width
Palate-Man1	Palate length/Mandible length1
Facial1-Man1	Facial length1/Mandible length1
Palate-Man2	Palate length/Mandible length2
Facial2-Man2	Facial length2/Mandible length2

Statistical analysis, including hypothesis testing, was done using IBM SPSS Statistics for Windows, Version 23.0 (IBM Corporation, New York, New York, USA). Results were interpreted as moderately significant (*p*-value < 0.1), significant (*p*-value < 0.05), and highly significant (*p*-value < 0.01).

## Results

The five healthy cheetah cubs comprised of two female (F662, F669) and three male cubs (M661, M668, M673). They were born to two different litters, but within 2 weeks of each another. All cubs were raised in the same institution with the same feeding regime. At the time of evaluation all cubs appeared clinically healthy and their body scores were appropriate for their age. Initially we intended to use four male and four female cubs, but in the following 2 years after the project started no litters were born at the facility. Therefore a decision was made to use the available data.

The results of the measurements obtained on each cheetah cub skull at each of the four evaluation times, including standard deviations, medians and 1st and 3rd quartiles, are given in Table 4. All length and width measurements of the skull and mandible showed similar trends with a marked increase in length/width between the 7 and 13th month observations whereafter growth rate stabilized. Maxillary width did not follow this pattern with the growth rate between the 7th to 13th month and 13th to 20 month very similar.

**Table 4:** Presentation of the descriptive statistics for each of the 13 skull measurements for the five cheetah cubs at each age interval.

**Description of measurement**	**Descriptive statistics**	**Age (months)**			
		**7**	**13**	**20**	**25**
Condylobasal length	Mean	140.79	169.76	181.74	180.90
	Std. Deviation	1.14	2.793	5.92	2.56
	Q1	140.16	167.70	179.651	179.50
	Median	141.22	169.04	183.33	181.31
	Q3	141.70	170.42	184.94	182.31
Palatal length	Mean	65.29	77.05	86.39	83.74
	Std. Deviation	0.91	1.84	1.64	1.50
	Q1	64.67	75.95	85.31	82.52
	Median	65.72	76.08	87.33	83.68
	Q3	65.95	78.25	87.54	85.20
Total skull length	Mean	156.86	189.45	201.14	200.74
	Std. Deviation	2.14	4.00	6.38	3.96
	Q1	154.96	187.38	199.81	199.70
	Median	157.34	188.22	200.39	200.90
	Q3	157.82	190.19	205.79	201.193
Facial length1	Mean	72.75	92.77	101.36	101.69
	Std. Deviation	1.53	2.48	4.86	2.57
	Q1	71.50	91.53	99.81	100.10
	Median	72.59	92.29	104.29	101.29
	Q3	74.29	93.25	104.54	104.00
Facial length2	Mean	51.27	63.63	67.63	70.34
	Std. Deviation	1.713	4.20	4.56	1.15
	Q1	50.49	61.54	64.24	69.78
	Median	50.77	63.23	67.31	70.00
	Q3	51.93	63.87	70.17	70.01
Neurocranium	Mean	88.46	99.18	104.55	104.45
	Std. Deviation	2.24	1.33	4.07	3.47
	Q1	86.97	98.05	104.26	103.28
	Median	88.51	99.22	105.70	104.48
	Q3	88.60	100.19	106.28	105.77
Maxilla width	Mean	72.36	77.55	81.80	79.14
	Std. Deviation	3.38	2.93	2.58	2.71
	Q1	70.25	75.88	80.21	77.69
	Median	72.87	77.67	80.81	78.33
	Q3	75.41	77.96	82.88	79.47
Skull width	Mean	109.55	130.28	138.15	133.77
	Std. Deviation	1.35	3.69	2.83	3.64
	Q1	108.91	128.79	136.41	131.93
	Median	109.25	128.93	136.63	132.45
	Q3	109.91	132.03	138.49	136.50
Mandible length1	Mean	93.83	116.72	124.80	101.00
	Std. Deviation	2.16	2.18	5.09	1.97
	Q1	93.05	116.01	125.73	99.40
	Median	94.35	116.26	126.74	100.79
	Q3	95.52	118.25	127.28	101.70
Mandible length2	Mean	77.10	93.57	100.60	126.99
	Std. Deviation	1.19	1.97	2.90	2.23
	Q1	76.34	91.62	100.29	125.94
	Median	76.35	93.96	100.68	126.84
	Q3	77.63	95.23	102.35	128.26
Mandibular height	Mean	19.79	20.68	21.61	22.55
	Std. Deviation	0.47	1.18	0.89	0.90
	Q1	19.59	19.75	21.70	21.65
	Median	19.98	20.74	21.7	22.79
	Q3	19.98	21.55	21.98	23.26
Molar height	Mean	0	11.5	11.8	11.3
	Std. Deviation	0	0.71	0.42	0.82
	Q1	0	11	11.75	10.75
	Median	0	12	12	11.5
	Q3	0	12	12	12
Molar angle	Mean	63.20	77.60	77.20	78.20
	Std. Deviation	6.38	3.71	3.90	4.76
	Q1	59.00	79.00	76.00	77.00
	Median	59.00	79.00	79.00	78.00
	Q3	68.00	79.00	79.00	82.00

*All measurements (excluding molar angle) were done in millimeters. Molar angle is given in degrees*.

Mandible height did not show a similar acceleration between 7 and 13 months. There was a more gradual increase in height over the 18-month period of evaluation (Table 4).

Eruption angles of the mandibular molar at the four observation times are also given in Table 4. Using Friedman's test we show that there was a significant difference in eruption angles of the five cheetah cubs evaluated (*p-value* = 0.017). Further evaluation using Dunn's multiple comparison indicated a moderately significant difference (*p-value* = 0.086) between the molar angles at age 7 months (molar unerupted but visible) compared to all the other observation times (7 vs. 13 months, 7 vs. 20 months, and 7 vs. 25 months) where the molars were fully erupted. No significant change in the molar angle occurred during the last three observations.

A total of 13 indices was calculated in order to quantify the neurocranium and facial growth in the five cheetah cubs during the 18 months of the study. The index values as well as the means, medians, standard deviations and 1st and 3rd quartiles are given in Table 5.

**Table 5:** The descriptive statistics for each of the 13 calculated indices for the skull measurements of five cheetah cubs at each age interval.

**Description of index**	**Descriptive statistics**	**Age (months)**			
		**7**	**13**	**20**	**25**
Palate length: Condylobasal length (Palate1)					
	Mean	0.464	0.454	0.475	0.463
	Std. Deviation	0.009	0.009	0.011	0.006
	Q1	0.456	0.449	0.468	0.463
	Median	0.464	0.450	0.474	0.465
	Q3	0.471	0.454	0.476	0.466
Palate length: Total skull length (Palate2)					
	Mean	0.416	0.407	0.430	0.417
	Std. Deviation	0.010	0.014	0.011	0.006
	Q1	0.411	0.403	0.420	0.413
	Median	0.412	0.406	0.425	0.416
	Q3	0.426	0.411	0.438	0.422
Facial length1:					
Condylobasal length(Facial1)					
	Mean	0.517	0.546	0.557	0.562
	Std. Deviation	0.007	0.007	0.011	0.008
	Q1	0.514	0.541	0.556	0.555
	Median	0.514	0.547	0.557	0.564
	Q3	0.524	0.552	0.565	0.565
Facial length1: Total skull length (Facial2)					
	Mean	0.464	0.490	0.504	0.507
	Std. Deviation	0.005	0.012	0.012	0.007
	Q1	0.460	0.485	0.499	0.504
	Median	0.461	0.486	0.501	0.504
	Q3	0.465	0.492	0.509	0.504
Facial length2: Condylobasal length (Facial3)					
	Mean	0.364	0.375	0.372	0.389
	Std. Deviation	0.010	0.020	0.015	0.007
	Q1	0.359	0.368	0.362	0.385
	Median	0.360	0.375	0.364	0.387
	Q3	0.366	0.377	0.383	0.395
Facial length2: Total skull length (Facial4)					
	Mean	0.327	0.336	0.336	0.350
	Std. Deviation	0.007	0.025	0.014	0.009
	Q1	0.322	0.327	0.326	0.346
	Median	0.327	0.341	0.336	0.349
	Q3	0.330	0.341	0.337	0.358
Facial length1: Neurocranium (Facial5)					
	Mean	0.823	0.935	0.969	0.974
	Std. Deviation	0.018	0.024	0.016	0.027
	Q1	0.807	0.919	0.957	0.953
	Median	0.822	0.931	0.960	0.969
	Q3	0.840	0.937	0.985	0.989
Facial length2: Neurocranium (Facial6)					
	Mean	0.580	0.641	0.647	0.674
	Std. Deviation	0.010	0.043	0.029	0.027
	Q1	0.570	0.630	0.637	0.660
	Median	0.585	0.634	0.639	0.666
	Q3	0.587	0.645	0.646	0.701
Maxilla width:					
Skull width(Maxilla-Skull width)					
	Mean	0.660	0.595	0.592	0.592
	Std. Deviation	0.027	0.017	0.016	0.008
	Q1	0.645	0.589	0.581	0.589
	Median	0.674	0.589	0.589	0.591
	Q3	0.677	0.602	0.591	0.591
Palate length: Mandible length1 (Palate-Man1)					
	Mean	0.696	0.660	0.693	0.829
	Std. Deviation	0.022	0.006	0.029	0.016
	Q1	0.688	0.656	0.688	0.825
	Median	0.695	0.661	0.692	0.832
	Q3	0.700	0.662	0.695	0.834
Facial length 1: Mandible length1 (Facial1-Man1)					
	Mean	0.776	0.795	0.812	0.801
	Std. Deviation	0.020	0.017	0.021	0.014
	Q1	0.758	0.787	0.807	0.794
	Median	0.778	0.792	0.823	0.795
	Q3	0.790	0.804	0.825	0.801
Palate length: Mandible length2 (Palate-Man2)					
	Mean	0.847	0.824	0.859	0.659
	Std. Deviation	0.022	0.030	0.030	0.009
	Q1	0.847	0.808	0.855	0.652
	Median	0.850	0.818	0.871	0.656
	Q3	0.861	0.832	0.871	0.665
Facial length 2: Mandible length2 (Facial2-Man2)					
	Mean	0.665	0.680	0.672	0.554
	Std. Deviation	0.028	0.037	0.040	0.014
	Q1	0.643	0.655	0.651	0.542
	Median	0.662	0.664	0.669	0.554
	Q3	0.680	0.697	0.700	0.564

**Figure 5:**
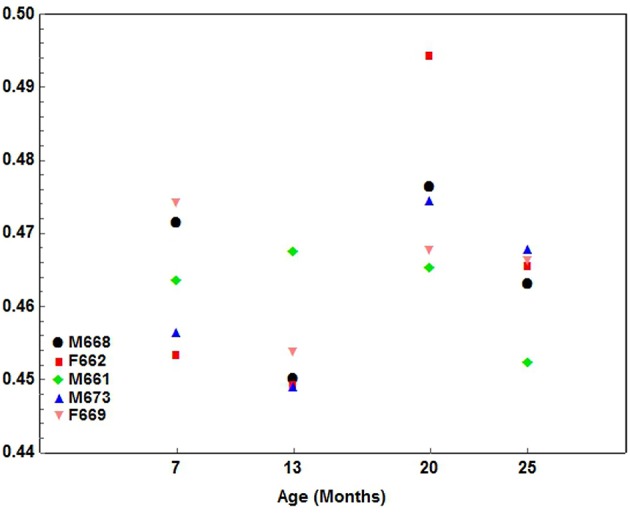
A scatterplot showing the development pattern of the Palate1 index (Palate length/Condylobasal length) in 5 cheetah cubs from 7 months-of-age at ~6-month intervals until they were 25 months-of-age. Each color presents a different cub as indicated.

**Figure 6:**
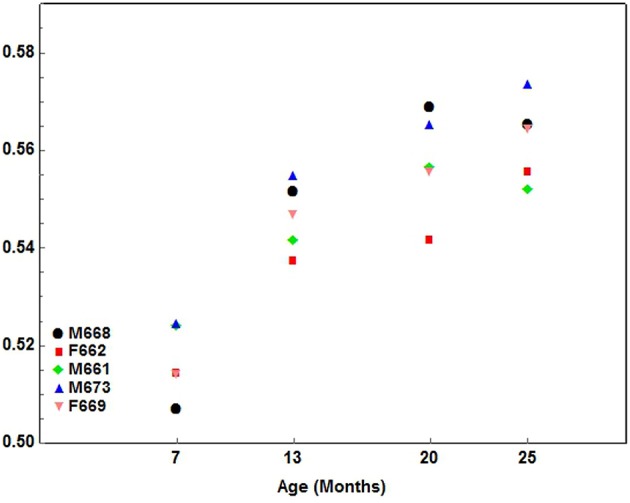
A scatterplot showing the development pattern of the Facial1 index (Facial length1/Condylobasal length) in the 5 cheetah cubs from 7 months of age at ~6-month intervals until they were 25 months of age. Each color presents a different cub as indicated.

From these indices two patterns of development were evident:

1. An alternating pattern where the one component of the index developed quicker for one interval, followed by another interval where the second component to the index predominated, and ending with a final interval where the initial component predominated. This type of development occurred for the following indices: Palate1 (Figure 5), Palate2, Palate-Man1 and Palate-Man2.

2. A gradual pattern where one component of the index developed quicker at every interval measured. The gradual pattern either had a positive (increasing) or negative (decreasing) slope. This type of development characterized by a gradual increasing pattern was seen in the following indices: Facial1 (Figure 6), Facial2, Facial3, Facial4, Facial5, and Facial6.

**Figure 7:**
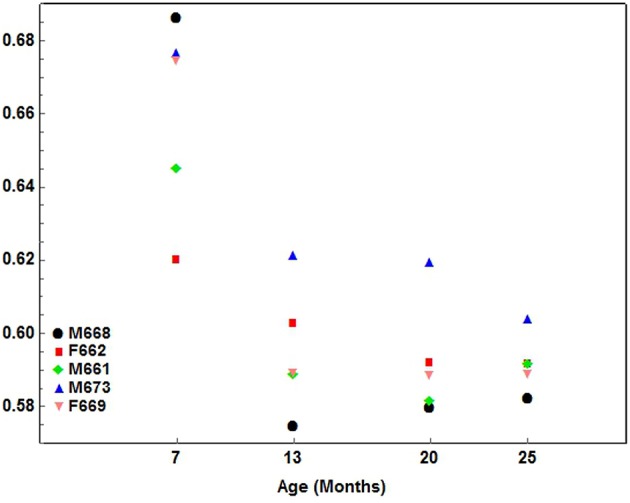
A scatterplot showing the development pattern of the Max—Skull width index (Maxilla width / Skull width) in the 5 cheetah cubs from 7 months of age at ~6-month intervals until they were 25 months of age. Each color presents a different cub as indicated.

A similar gradual pattern of development, but with a negative inclination (decreasing), was seen for Maxilla:Skull width only (Figure 7).

Seven of the indices measured showed significant differences between the different evaluation times. The outcomes of Friedman's test and Dunn's multiple comparisons in testing for differences between these indices are given in Table 6.

**Table 6:** Listing of the seven calculated indices for cranium and facial growth in five young growing cheetahs which were significantly different from each other using the Friedman's test, followed by the significance levels using Dunn's multiple comparisons test.

**Index calculated**		**Dunn's multiple comparison (*****p*****)**					
	**Friedman**	**7 vs. 13**	**7 vs. 20**	**7 vs. 25**	**13 vs. 20**	**13 vs. 25**	**20 vs. 25**
Facial length1: Condylobasal length	0.004^***^	1.000	0.020^**^	0.009^***^	0.518	0.300	1.000
Facial length1: Total skull length	0.004^***^	1.000	0.020^**^	0.009^***^	0.518	0.300	1.000
Facial length2: Total skull length	0.033^**^	0.519	0.518	0.020^**^	1.000	1.000	1.000
Maxilla width: Skull width	0.019^**^	1.000	1.000	0.020^**^	1.000	0.086^*^	0.300
Palate length1: Mandible length1	0.003^***^	0.850	0.165	0.001^***^	1.000	0.165	0.850
Palate length: Mandible length2	0.014^**^	0.518	0.042^**^	0.020^**^	1.000	1.000	1.000
Facial length2: Mandible length2	0.026^**^	0.165	0.086^*^	0.042^**^	1.000	1.000	1.000

* *moderately significant (p-value < 0.1)*,

** *significant (p-value < 0.05)*,

*** *highly significant (p-value < 0.01)*.

## Discussion

Longitudinal studies work best with large sample sizes. However, generating data from large sample sizes of an endangered species like cheetah for a longitudinal study poses significant challenges. As a result, we described the findings for this small group of cheetah, mindful that studies using larger cheetah numbers could possibly produce different results.

In the five young cheetah from the Ann van Dyk cheetah center, the cranio-maxillofacial parameters measured closely follows the skeletal development pattern (1). The maximum growth rate for all cranio-maxillofacial measurements was reached within the 7 to 13 month interval. Maxilla width and mandibular height were the only exception to the growth acceleration recorded during this interval.

In an attempt to describe the growth rate of the various components of the maxillo-facial complex, a series of indices was calculated. From these it was clear that different components of the maxillo-facial complex developed at different rates. The rate of development of the palate, compared to both the condylobasal and total skull lengths, fluctuates and appears to have a faster development than either the aforementioned recorded during the 13 to 20 month interval. A similar pattern of development occurred when comparing the palate length with both mandibular lengths measured for the mandible.

Facial development seems to progress at a higher rate than condylobasal -, total skull - or neurocranium development. This follows a positive near linear relationship (this study). The development of the facial structures, especially those associated with the respiratory and alimentary tracts, as well as the mandible, follows a somatic growth pattern. This is in contrast to the brain, skull, eyeballs, etc., which predominantly follows a neural growth pattern which allows for maximal size to be obtained early in life (12).

Most development in the maxillofacial complex occurs in a rostro-caudal direction with very little development occurring in the width of the skull. This is due to this growth in the maxillofacial complex being only dependent on the intranasal, intermaxillary and median palatal sutures as well as the mandibular symphysis (12). This was indeed the case with our five cheetah where the maxillary width developed slower than the skull width. In addition, a gradual decreasing trend was evident in the index comparing maxilla width to skull width over an 18-month period.

The molar angle changed very little once the tooth was fully erupted. Just prior to eruption (7 months of age) the angle of the molar compared to the ventral mandible is more acute than once erupted. The fact that these captive born cheetah had no significant change in their molar angle over time is significant (this study) and does not support the theory of molar angulation changes to be the cause of FP (previously known as FPE) in the palatine indentations associated with it (3).

The skulls and maxillo-facial complex of the five growing cheetah followed the same neural and somatic growth patterns as have been described before in other mammals, excluding humans and great apes where a pubertal growth spurt of the condylobasal occurs (12). Development in the rostrocaudal direction is more evident than growth in width which again follows the general pattern seen in mammals, excluding humans and the great apes, where a superior/inferior (dorso-ventral) increase in height is more common (12).

Eruption of the mandibular molar teeth occurs after 7 months of age and is in full occlusion by 13 months. Once erupted the molars do not change their angulation. This is in keeping with the unique fact that in the specialized dentition of carnivores teeth do not super erupt or drift once contact with opposing or adjacent teeth is lost (13). The results of the present study could, therefore, not confirm an influence of a captive diet on the position or angulation of the mandibular molars in cheetah.

## Ethics Statement

The study was approved by the University of Pretoria's Animal Ethics Committee (Project: EC062-11).

## Author Contributions

GS: design of project, acquisition and analysis, interpretation, writing, and editing of manuscript. MB and MS: design of project, writing, and editing of manuscript. PvS: design of project, statistical analyses, interpretation, writing, and editing of manuscript.

### Conflict of Interest Statement

The authors declare that the research was conducted in the absence of any commercial or financial relationships that could be construed as a potential conflict of interest.
